# Adipocire

**Published:** 1847-04

**Authors:** 


*Adipocire.*—By the courtesy of our friend Dr. Blakeman, of Bleecker street, we were invited, a few days since, in company with

Drs. Miner, Senr., J. K. Rodgers, A. C. Post, Linsley, F. C. Stewart, Borrowe, Williams, Rockwell, A. E. Hosack, &c., to inspect the body of a Mrs. F., which had been disinterred from a burying ground in Twelfth street, in which it had lain for 17 years. It was that of a very large woman. The coffin in which it had been enclosed was very little affected by its inhumation. The form of the cadaver was perfectly preserved. The extremity of the nose was gone, and the features of the face partially discernible. The arms, which were placed along the sides, had become much compressed by the swelling of the body, and the hands had broken off at the wrists. The remains of one hand, upon the right side of the abdomen, were distinctly visible, and the forefinger, with its nail, was entire. The rotundity of the breast was perfect—the abdomen flattened, as if by compression against the lid of the coffin. The left foot, like the hands, had separated from the ankle. The rotundity and shape of the thighs was remarkably preserved. The shroud adhered to the body and was not decayed, though discoloured. The cadaver presented a greenish hue from mould, with which it was covered, and a fresher coat of pure white, had been deposited upon it since its exposure to the air. The cap was distinct upon the head, and the bow of black ribbon on one side, somewhat faded, remained. By the consent of a son of the deceased, who was in possession of the body, a piece about a foot square was cut out of the abdominal walls, by Dr. Post, so as to expose the cavity. The knife passed easily through their substance, which was found to consist entirely of a yellowish cheesy substance. The thin layer of abdominal muscles could be discriminated, similarly converted beneath the adipose layer, which was very thick. The inner surface of the abdominal cavity was smooth. The viscera and a large lump of adipocire, lay at the bottom. The diaphragm was distinctly seen. Below it, and towards the left, much shrunken and condensed, and having the consistence, somewhat, of lung, lay the liver. The stomach, having its natural form and a macerated appearance, was distinctly visible, and some portions of the colon, collapsed and membranous, were discoverable. This was all that could be seen within the abdominal cavity through the aperture that was made. The exsected piece of abdominal wall was then replaced. An odour closely resembling that of gum ammoniac, exhaled from the body. We learn that the soil in which the coffin was interred, was sandy, and not moist; and that two bodies of children buried over it, and others in the vicinity, were in a state of complete disorganization. Some finger-bones, lying loose in the coffin, were taken up, and found so much softened as to be cut easily, but not altered in form. The shavings which the coffin contained, were blackened, but otherwise unaltered. The subject was 69 years of age, very fat, and weighed 170 pounds. The disease of which she died has escaped our recollection. The conversion of the human body into adipocire, though rare among us, has been not unfrequently met with. A body in this state of preservation was exhibited some time ago, at one of our public museums, coming, we believe, from Canada.—*N. Y. Annalist.*

